# Neuroprotective effects on microglia and insights into the structure–activity relationship of an antioxidant peptide isolated from *Pelophylax perezi*

**DOI:** 10.1111/jcmm.17292

**Published:** 2022-04-23

**Authors:** Alexandra Plácido, Constança do Pais do Amaral, Cátia Teixeira, Ariane Nogueira, José Brango‐Vanegas, Eder Alves Barbosa, Daniel C. Moreira, Amandda É. Silva‐Carvalho, Maria da Gloria da Silva, Jhones do Nascimento Dias, Patrícia Albuquerque, Felipe Saldanha‐Araújo, Filipe C. D. A. Lima, Augusto Batagin‐Neto, Selma Kuckelhaus, Lucinda J. Bessa, Jaime Freitas, Guilherme Dotto Brand, Nuno C. Santos, João B. Relvas, Paula Gomes, José Roberto S. A. Leite, Peter Eaton

**Affiliations:** ^1^ 26706 Department of Chemistry and Biochemistry LAQV/REQUIMTE Faculty of Sciences University of Porto Porto Portugal; ^2^ 37809 Instituto de Medicina Molecular Faculdade de Medicina Universidade de Lisboa Lisbon Portugal; ^3^ 28127 Center for Research in Applied Morphology and Immunology (NuPMIA) University of Brasilia Brasilia Brazil; ^4^ Laboratory of Synthesis and Analysis of Biomolecules (LSAB) Institute of Chemistry (IQ) University of Brasilia Brasília Brazil; ^5^ 28127 Laboratory of Hematology and Stem Cells Faculty of Health Sciences University of Brasilia Brasília Brazil; ^6^ Department of Cell Biology Institute of Biological Sciences University of Brasília Brasília Brazil; ^7^ Biomedicine Course Federal University of Delta do Parnaíba (UFDPar) Parnaíba Brazil; ^8^ Faculty of Ceilândia University of Brasilia Brasilia Brazil; ^9^ Federal Institute of Education, Science and Technology of São Paulo Matão Brazil; ^10^ São Paulo State University (UNESP) Campus of Itapeva Itapeva SP Brazil; ^11^ Egas Moniz Interdisciplinary Research Center (CiiEM) Egas Moniz ‐ Cooperative for Higher Education CRL Almada Portugal; ^12^ Institute for Research and Innovation in Health (i3S) National Institute of Biomedical Engineering (INEB) University of Porto Porto Portugal; ^13^ Institute for Research and Innovation in Health (i3S) Institute for Molecular and Cell Biology (IBMC) University of Porto Porto Portugal; ^14^ The Bridge School of Chemistry Joseph Banks Laboratories University of Lincoln Lincoln UK

**Keywords:** Amphibia, antioxidant, bioactive peptide, neuroprotection, *Pelophylax perezi*, tryptophyllin

## Abstract

Tryptophyllins constitute a heterogeneous group of peptides that are one of the first classes of peptides identified from amphibian’s skin secretions. Here, we report the structural characterization and antioxidant properties of a novel tryptophyllin‐like peptide, named PpT‐2, isolated from the Iberian green frog *Pelophylax perezi*. The skin secretion of *P*.* perezi* was obtained by electrical stimulation and fractionated using RP‐HPLC. De novo peptide sequencing was conducted using MALDI MS/MS. The primary structure of PpT‐2 (FPWLLS‐NH_2_) was confirmed by Edman degradation and subsequently investigated using *in silico* tools. PpT‐2 shared physicochemical properties with other well‐known antioxidants. To test PpT‐2 for antioxidant activity *in vitro*, the peptide was synthesized by solid phase and assessed in the chemical‐based ABTS and DPPH scavenging assays. Then, a flow cytometry experiment was conducted to assess PpT‐2 antioxidant activity in oxidatively challenged murine microglial cells. As predicted by the in silico analyses, PpT‐2 scavenged free radicals *in vitro* and suppressed the generation of reactive species in PMA‐stimulated BV‐2 microglia cells. We further explored possible bioactivities of PpT‐2 against prostate cancer cells and bacteria, against which the peptide exerted a moderate antiproliferative effect and negligible antimicrobial activity. The biocompatibility of PpT‐2 was evaluated in cytotoxicity assays and *in vivo* toxicity with *Galleria mellonella*. No toxicity was detected in cells treated with up to 512 µg/ml and in *G*.* mellonella* treated with up to 40 mg/kg PpT‐2. This novel peptide, PpT‐2, stands as a promising peptide with potential therapeutic and biotechnological applications, mainly for the treatment/prevention of neurodegenerative disorders.

## 1 INTRODUCTION

The skin is one of the most stress‐exposed body tissues and requires certain mechanisms to cope with such pressure.^1^, ^2^ Amphibians’ skin presents an arsenal of original bioactive compounds^3^ that allows adaptation to environments with extreme features, such as high oxygen levels,^4^ low temperature and high ultraviolet (UV) radiation. Additionally, the skin secretion of most amphibians is composed by molecules that act in a variety of defence mechanisms against external aggressors, such as microorganisms, parasites or predators.^5^, ^6^

Tryptophyllins are one of the first classes of peptides identified from amphibian’s skin secretions.^7^ Most tryptophyllins have a tryptophan residue at position 2 from the *C*‐terminus and one or two prolines at positions 2 and 3 from the *N*‐terminus.^7^ As tryptophyllins are a heterogeneous group of peptides, they were recently reclassified into three groups: T‐1 (amidated heptapeptides and non‐amidated octapeptides having an *N*‐terminus formed by lysine (Lys) and prolyl (Pro) residues (Lys‐Pro), tryptophanyl (Trp) residue at position 5 and Pro residue at position 7), T‐2 (four to seven amino acids residues, having a common internal Pro‐Trp) and T‐3 (tridecapeptides with five conserved Pro and absence of Trp).^8^ Tryptophyllins have been commonly associated with myoactivity and vasorelaxation/vasoconstriction properties.^8^, ^9^, ^10^ Other relevant biological activities, such as opioid,^11^ antiproliferative^10^ and antimicrobial actions,^12^ have also been reported.

Thus, the roles of tryptophyllins as part of the arsenal of peptides from amphibian’s skin secretion remain uncertain, as all the biological activities described above are modest, leading to doubts about their physiological relevance. Exposing amphibian’s skin to UV radiation during daylight coupled with a high availability of O_2_ in the transition from aquatic to terrestrial habitats can lead to the generation of reactive oxygen species (ROS). Recently, peptides with antioxidant properties have been characterized from the skin secretion of amphibians, suggesting a physiological role of some peptides in counteracting oxidative stress.^13^, ^14^ Indeed, the first peptide expressed in the skin secretion of *Pithecopus azureus* along its metamorphosis, when it shifts from the water to land, has antioxidant activity.^15^

Amphibian antioxidant peptides (AOPs) have shown ROS‐scavenging properties, being identified in a variety of genera. The majority of them are found in genera from the Ranidae family, such as *Hylarana*, *Amolops*,^16^ *Rana* ^2^ and *Odorrana*,^4^ but also occur in genera from other families, such as *Nanorana* (Dicroglossidae)^17^ and *Physalaemus* (Leptodactylidae),^13^ with high structural variability.

In this work, a novel amidated peptide named PpT‐2 was identified from the cutaneous secretion of the Iberian green frog (*Pelophylax perezi*) from São Miguel Island in the Azores archipelago, Portugal. Morphological and histological details of the skin were investigated to identify the accessory and granular glands. The possible relationship between PpT‐2 and oxidative protection was tested *in silico* and through *in vitro* radical scavenging assays. To the best of our knowledge, we have identified for the first time a tryptophyllin‐like peptide with antioxidant potential.

In addition, *in silico* studies were carried out to evaluate proline transition and its relationship with physicochemical properties, as well as antioxidant properties and local reactivities of PpT‐2 in relation to other compounds. Cytotoxicity studies in mammalian models, including against human prostate cancer cells (PC3 cell line) and *in vivo* toxicity in *G*. *mellonella* larvae, were conducted. Finally, ROS and reactive nitrogen species (RNS) were quantified with intracellular fluorescent probes in BV‐2 microglial cells to assess the neuroprotective potential of PpT‐2.

## 2 MATERIAL AND METHODS

### 2.1 Purification, characterization and sequence analysis

The dry secretion (1 mg) was dissolved in Milli‐Q water (500 μl) and subjected to separation in an HPLC system (LC‐20 CE; Shimadzu), using a Vydac C_18_ reverse‐phase column (2018 TP). The fractions were eluted with a linear gradient of 0.1% (v/v) aqueous trifluoroacetic acid/acetonitrile, ranging from 5% to 60% over 60 min, and 75–95% over 5 min, at a flow rate of 1 ml/min. Fractions were monitored at 216 and 280 nm, collected in tubes and dried under vacuum centrifugation. Details of the collection of biological material and sample processing for morphological studies of the skin are in the Supplementary Material. Furthermore, aliquots were prepared for mass spectrometry (MALDI‐TOF/TOF) and Edman degradation, as explained in detail in the Supplementary Material.^18^

### 2.2 Computational studies: *cis*‐*trans* isomerization and antioxidant properties

PpT‐2 sequence was designed with the aid of the Avogadro computational package, accounting for the amide modification on the *C*‐terminus and protonation of the *N*‐terminus.^19^ Preliminary conformational evaluations were conducted via molecular dynamics (MD) simulations at high temperature, to accurately reproduce structural features of the peptide.^20^ For this purpose, PpT‐2 was placed in contact with a thermal reservoir at 1000 K, and 100 distinct conformations were stored during the dynamics for subsequent geometry optimization (50 structures with initial *cis* conformation of proline and 50 *trans*). MD conformational searches were conducted using AMBER force field with the aid of the Gabedit computational package.^21^, ^22^ Preliminary geometry optimizations were conducted for all the conformers in a Hartree–Fock (HF) approach and optimized in the framework of Kohn–Sham density functional theory (KS‐DFT). Condensed‐to‐atoms Fukui indices (CAFI), local softness and donor‐acceptor maps (DAM) were calculated to predict the local reactivity of the sequence. All the *in silico* approaches are described in detail in the S1.^23^, ^24^, ^25^, ^26^, ^27^, ^28^ The antioxidant properties of PpT‐2 were compared to those of salamandrin‐I (protonated and non‐protonated structures), glutathione, trolox and other antioxidant compounds, estimated at the same level of theory.

### 2.3 Peptide synthesis and quantification

During this work, two methodologies of solid‐phase peptide synthesis (SPPS) were used (see Supplementary Material for details).^29^, ^30^, ^31^, ^32^, ^33^

### 2.4 Antibacterial and in vitro radical scavenging assays

The antibacterial activity of PpT‐2 was assessed using the broth microdilution method according to the recommendations of the Clinical and Laboratory Standards Institute^34^ and as previously described,^35^ against four reference strains, namely *Escherichia coli* ATCC 25922, *Pseudomonas aeruginosa* ATCC 27853, *Staphylococcus aureus* ATCC 29213, and *Enterococcus faecalis* ATCC 29212. The peptides were tested in the concentration range of 1–1024 μg/ml.

Free radical scavenging activity was assessed *in vitro* using two chemical‐based assays: the ABTS (2,2‐azino‐bis(3‐ethylbenzothiazoline‐6‐sulphonic acid) and DPPH (2,2‐diphenyl‐1‐picrylhydrazyl) assays. For both assays, PpT‐2 was diluted in phosphate buffer saline (PBS) containing 27% (v/v) dimethylsulfoxide (DMSO) to prepare the stock solution at 1.5 mg/ml. The stock solution was diluted to several concentrations (0.031–0.25 mg/ml) using PBS prior to the assays.^16^, ^36^, ^37^

### 2.5 ROS and RNS intracellular analysis by flow cytometry assay

Reactive oxygen species and RNS were quantified with intracellular fluorescent probes in BV‐2 microglial cells (BCRJ, #0356) and SK‐N‐BE(2) (*Homo sapiens*, tissue brain; neuroblast, CRL‐2271‐ATCC). Intracellular ROS production was measured using DCFH‐DA (Sigma‐Aldrich) and that of RNS was measured using DAF‐FM (Sigma‐Aldrich). Cells (BV‐2 and SK‐N‐BE(2)) were adhered to the plate for 2 h (1.0 × 10^5^ cells/well; 200 μl/well) and then subjected to the following treatments: DMEM medium; 100 nM PMA (phorbol 12‐myristate 13‐acetate) (Sigma‐Aldrich); 100 nM PMA + 50 μM PpT‐2; 100 nM PMA + 100 μM PpT‐2; 50 μM PpT‐2; or 100 μM PpT‐2 for 30 min (BV‐2 cells) and 60 min (Sk‐n‐be (2) cells). Then, probes (DAF‐FM and DCFDA) were added according to the manufacturer's instructions. ROS and RNS production was evaluated by flow cytometry (FACSCalibur; BD Bioscience), with excitation at 488 nm. Emission was detected in the FL‐1 (515–545 nm) channel. Ten thousand events were recorded for each sample, and data were analysed using FlowJo v. 10.7.

### 2.6 Cytotoxicity assays in human cells

#### 2.6.1 Haemolysis assay in human blood cells

The haemolytic activity of PpT‐2 was tested using human red blood cells (RBCs), mostly as previously described,^38^ except some modifications. Briefly, human blood samples were collected in K_3_EDTA (ethylenediaminetetraacetic acid, potassium salt) collection tubes; RBCs were isolated by centrifugation and washed three times with 50 mM phosphate‐buffered saline (PBS) pH 7.4. Peptide solutions of increasing concentrations (0–400 µM) were added to 1% (v/v) RBCs suspensions and incubated for 1 h at 37°C. Samples were then centrifuged at 870 × *g* for 5 min. The supernatants were collected, and the absorbance (A) at 540 nm was measured. Positive control (100% haemolysis) was achieved by adding 0.1% Triton X‐100 (v/v) to a sample, and negative control was determined for a RBCs suspension in PBS pH 7.4. Haemolysis assays were performed in triplicates. Haemolysis percentage was calculated as: [(A(peptide)‐A(PBS)/(A(Triton X‐100) – A(PBS)] × 100.^18^, ^38^, ^39^, ^40^

#### 2.6.2 Cell viability assay of a human microglial cell line

The cytotoxicity of PpT‐2 was also assessed using microglial human cells. The human microglial cell line HMC3 was obtained from ATCC (ATCC^®^ CRL3304TM). These cells were cultured with DMEM GlutaMAX™‐I (Thermo Fisher Scientific) supplemented with 10% foetal bovine serum (FBS) (Thermo Fisher Scientific), 100 U/ml penicillin and 100 μg/ml streptomycin (Thermo Fisher Scientific) and maintained at 37°C, 95% air and 5% CO_2_ in a humidified incubator.

Cell viability was determined by measuring total cellular metabolic activity using the reduction in resazurin to the fluorescent resorufin. Briefly, following 24 h of exposure to PpT‐2, 8 μl of a 400 μM resazurin solution was added to each well. After 4 h of incubation in the dark (37°C; 95% air/5% CO_2_), fluorescence was measured by fluorescence spectrometer at λ_excitation_ = 530 nm and λ_emission_ = 590 nm. All exposures were performed in triplicate, and every assay was repeated in triplicate.^41^

#### 2.6.3 Cell viability assay of a human prostate cancer cell line

Human prostate cancer cell, PC3 (ATCC^®^ CRL‐1435™, *Homo sapiens* prostate; derived from metastatic site: bone), was cultured in RPMI medium (Sigma‐Aldrich) supplemented with 10% (v/v) FBS (Sigma‐Aldrich) and 1% (v/v) penicillin‐streptomycin (Lonza) at 37ºC and 5% CO_2_ atmosphere.

The sodium 3´‐[1‐(phenylaminocarbonyl)‐3,4‐tetrazolium]‐bis (4‐methoxy‐6‐nitro) benzene sulfonic acid hydrate (XTT) colorimetric cell proliferation kit (Sigma‐Aldrich) was used to assess cell viability,^42^ according to the manufacturer’s instructions. Briefly, PC3 cells were seeded in triplicates into 96 well‐plates at a cell density of 5 × 10^4^ cells/ml. After 24 h, cells were washed with PBS and treated with increasing concentrations of PpT‐2 (10–400 μM) diluted in non‐supplemented RPMI medium for another 48 and 72 h. The XTT mixture was added for an additional 4 h incubation. Absorbance (A) was measured at 492 nm with a reference wavelength at 690 nm, and cell viability was determined through the following equation: Cell viability (%) = ((A_492_–A_690_)_sample_/(A_492_–A_690_) _control_) × 100. Cell viability assays were performed in triplicate.^43^

### 2.7 *In vivo* toxicity in *Galleria mellonella* larvae

PpT‐2 toxicity was further evaluated using the *in vivo* model of *Galleria mellonella,* as described by Balasubramanian et al.,^44^ with some modifications. Groups of 16 similar‐size 6th instar larvae (250–300 mg) were injected with 10 μl of different doses of PpT‐2 (40, 20 and 10 mg/kg) diluted in PBS or with PBS alone (control group). After treatment, larvae were kept at 30ºC for 7 days and daily monitored for survival. The assay was performed at least twice, and the Kaplan–Meier survival curve was generated using GraphPad Prism 6 software.

## 3 RESULTS AND DISCUSSION

### 3.1 Morphology of the glands and identification of PpT‐2

**FIGURE 1:**
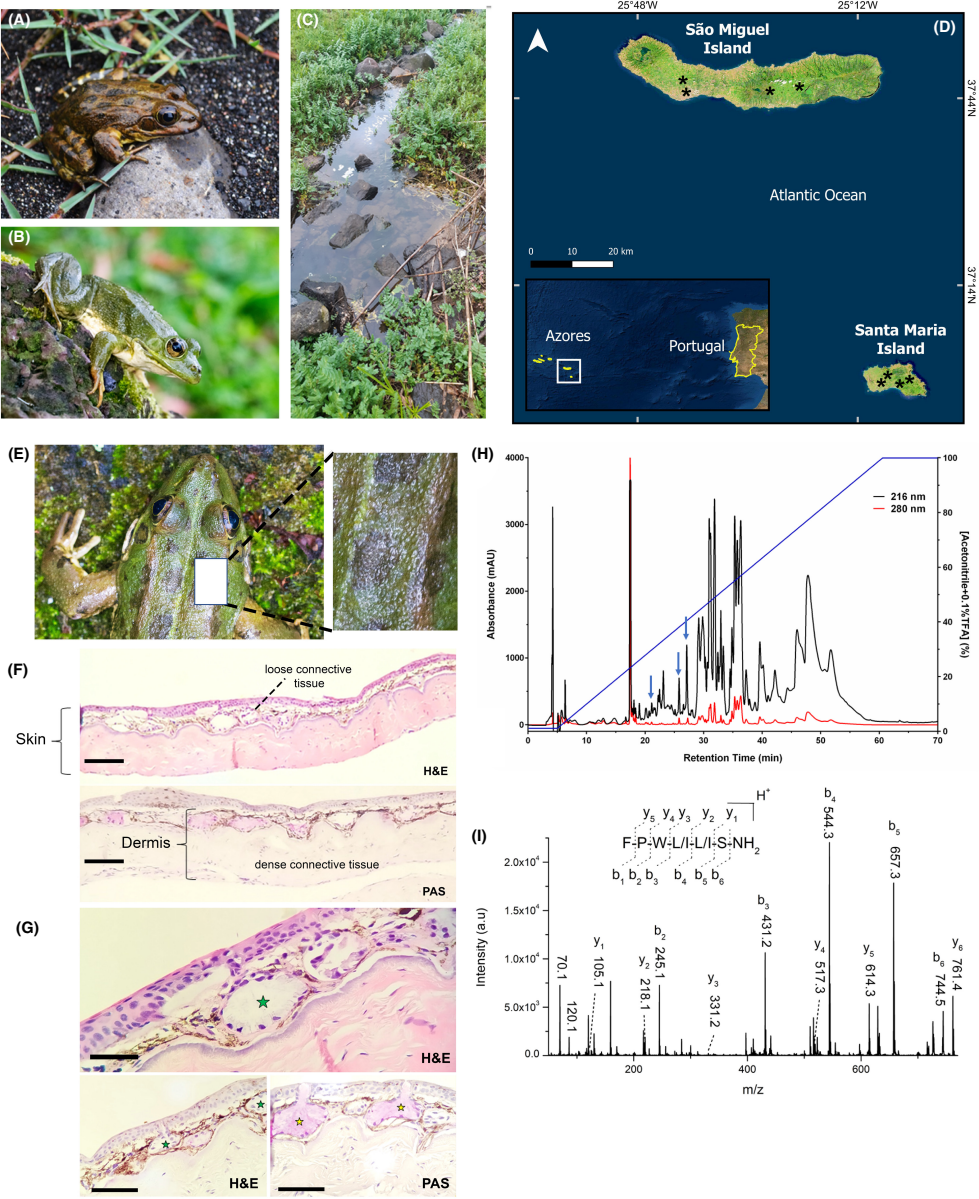
(A,B) Adult specimens of *Pelophylax perezi* (López‐Seoane 1885) showing polymorphism in skin pigmentation with different colour patterns. (Photos: Peter Eaton) (C) Typical habitat of *P*. *perezi* in the Azores archipelago is a small body of permanent water (Santa Maria Island, Azores, Portugal). (Photo: José Leite) (D) Distribution map of *P*. *perezi* collection in this work in the Azores archipelago (São Miguel and Santa Maria islands). (E) Detail of *P*. *perezi* showing the area of the dorsal region of the animal, with greater predominance of glandular tissue, where the histological analysis was performed. (F) Skin photomicrographs of *P*. *perezi*, showing the superficial epidermis and the dermis divided into a loose subepithelial layer and a deep dense layer. The epithelial tissue is of the stratified pavement [epithelial pavement (EP)] type formed by up to four cell layers (scale bar: 200 µm) (H&E: haematoxylin and eosin stain). (G) In the loose dermis, there are innumerable simple, serous (green star) and mucous (yellow star) alveolar glands and dense granulation of dispersed melanin (M) between the alveoli (F and G) (Figure 2C, bar: 200 µm). In the deep dermis, the collagen fibre bundles exhibit unmodified disposition and the fibroblast nuclei scattered in the extracellular matrix. Glycoproteins and mucopolysaccharides were labelled by Schiff reactive (Schiff periodic acid, PAS). (H) Reverse‐phase HPLC chromatogram of the crude extract from *P*. *perezi* skin secretion. Sample absorbance was monitored at 216 (black line) and 280 nm (red line) in arbitrary units (a.u.). The fractions containing PpT‐2 are indicated by the blue arrow. (I) MS/MS spectra of PpT‐2, [M+H]^+^=761.4 Da, acquired in an UltrafleXtreme MALDI‐TOF/TOF; amino acid sequence FPWL/IL/IS‐NH_2_

Tryptophyllins are a large but heterogenous family of peptides first isolated from the *Phyllomedusa* genus.^10^ However, structurally related peptides have been identified in other amphibian genera, including *Litoria* from Oceania.^45^ In our work, we identified a new tryptophyllin in the skin secretion of *P*. *perezi* (Figure 1A,B) from the Archipelago of the Azores (Figure 1C,D).

Notably, this is the first peptide identified in the skin secretion of *P*.* perezi*, in addition to being the first tryptophyllin identified in a species of distribution in Europe. The distribution of the *P*. *perezi* specie comprises the Iberian Peninsula and the south of France, and the northern limit of its distribution is probably south to the Loire basin.^46^

*Pelophylax perezi* is not endemic to the oceanic islands and, according to anthropological studies, it has been introduced to that habitat at least 200 years ago.^47^ Currently, it is distributed across all islands of the Azores archipelago, living in temporary bodies of water, such as lagoons formed by rains (Figure 1C). The skin secretions of many, but not all, anurans (frogs and toads) that contain bioactive peptides are stored in granular glands.

These glands are located mainly in the dorsal skin, where glands are surrounded by myocytes and innervated by sympathetic fibres.^48^ Details of *P*. *perezi* dorsal skin show a greater predominance of glandular tissue where the histological analyses were performed (details of the histological analysis are included in the Supporting information section) (Figure 1E). Superficial epidermis and the dermis are divided into a loose subepithelial layer and a deep dense layer (Figure 1F). The stratified squamous epithelium is formed by up to four cell layers (Figure 1G). In the loose dermis, there are innumerable simple, serous and mucous alveolar glands and dense granulation of dispersed melanin between the alveoli (Figure 1F,G). In the deep dermis, the collagen fibre bundles exhibit unmodified disposition and the fibroblast nuclei are scattered in the extracellular matrix.

Despite the large variety of peptides already described in the biodiversity of European amphibians,^40^ such as the antimicrobial brevinins^49^ and esculentins,^50^ no studies had been done with the skin secretion of *P*. *perezi*. From the HPLC analysis of the crude skin secretion, we identified more than 20 fractions, including that containing the peptide under investigation (Figure 1H).

This was detected as a novel component of the skin secretion, characterized by MALDI‐TOF MS and Edman degradation to possess a molecular mass of 761.41 Da and the amino acid residue sequence FPWLLS‐NH_2_ (Figure 1I). Sequence analysis allowed us to conclude that this was a tryptophyllin‐like peptide, named PpT‐2 to reflect the amphibian species of origin (*Pelophylax perezi*) and the tryptophyllin class 2.^9^

Chromatographic analysis showed the presence of at least three fractions containing PpT‐2, which could be explained by the presence of proline in its sequence. Prolines are associated with the presence of different *cis*/*trans* rotamers (*vd*. *infra*); this will be discussed further below, when addressing computational studies undertaken.

A comparison of the primary structure of PpT‐2 with those of other previously reported tryptophyllins is shown in Table 1. All members of this heterogenous family fall into three discrete groups (T‐1, T‐2 and T‐3). Tryptophyllins remain the only group of peptides isolated from amphibian skin secretions that are characterized solely on a chemical basis, rather than by specific bioactivity.^9^ For this reason, the bioactivity of this peptide group remains unclear and reference to their primary structures indicates a high degree of heterogeneity. Identified for the first time in Neotropical phyllomedusine frog skin,^10^ tryptophyllin‐2 peptides have been isolated from the skin secretion of the Australian tree frog *Litoria rubella*.^45^

**TABLE 1:** Primary structures, origin and physical‐chemistry parameters of amphibian tryptophyllins

Sequence	Name	Biology source	Geographic distribution	M_w_	pI	Reference
FPWLLSa	PpT‐2	*Pelophylax perezi*	São Miguel Island^a^, Azores	761.4	13.8	This work
DMSPPWHa	PdT‐2	*Pachymedusa dacnicolor*	Endemic to Mexico	876.98	7.8	[14]
FPPWV	T‐2a	*Phyllomedusa rhodei*	Endemic to Brazil	643.79	13.8	[9]
FPPWLa	T‐2b	*Phyllomedusa rhodei*	Endemic to Brazil	657.81	13.8	[9]
FPPWMa	T‐2c	*Phyllomedusa rhodei*	Endemic to Brazil	675.85	13.8	[9]
pQPWVa	T‐2d	*Phyllomedusa rhodei*	Endemic to Brazil	638.72	13.8	[9]
pQPWMa	T‐2e	*Phyllomedusa rhodei*	Endemic to Brazil	670.79	13.8	[9]
pQFPWL	L1	*Litoria rubella*	Oceania	799.93	13.8	[17]
FPWL	L2	*Litoria rubella*	Oceania	561.68	6.0	[17]
FLPWY	L3	*Litoria rubella*	Oceania	724.86	5.9	[17]
pQIPWFHR	L4	*Litoria rubella*	Oceania	1094.24	9.2	[17]
KP(HyP)AWPa	PdT‐1	*Pachymedusa dacnicolor*	Endemic to Mexico	709.85	13.9	[10]

Note

R‐a (C‐terminal amidation); pI, isoelectric point; M_w_, Molecular weight.

^a^ The distribution of the *P*. *perezi* comprises the Iberian Peninsula and the south of France, but the specimens of this work were collected on the Island of São Miguel, Azores.

### 3.2 Theoretical structural analysis on conformation of proline in PpT‐2

**FIGURE 2:**
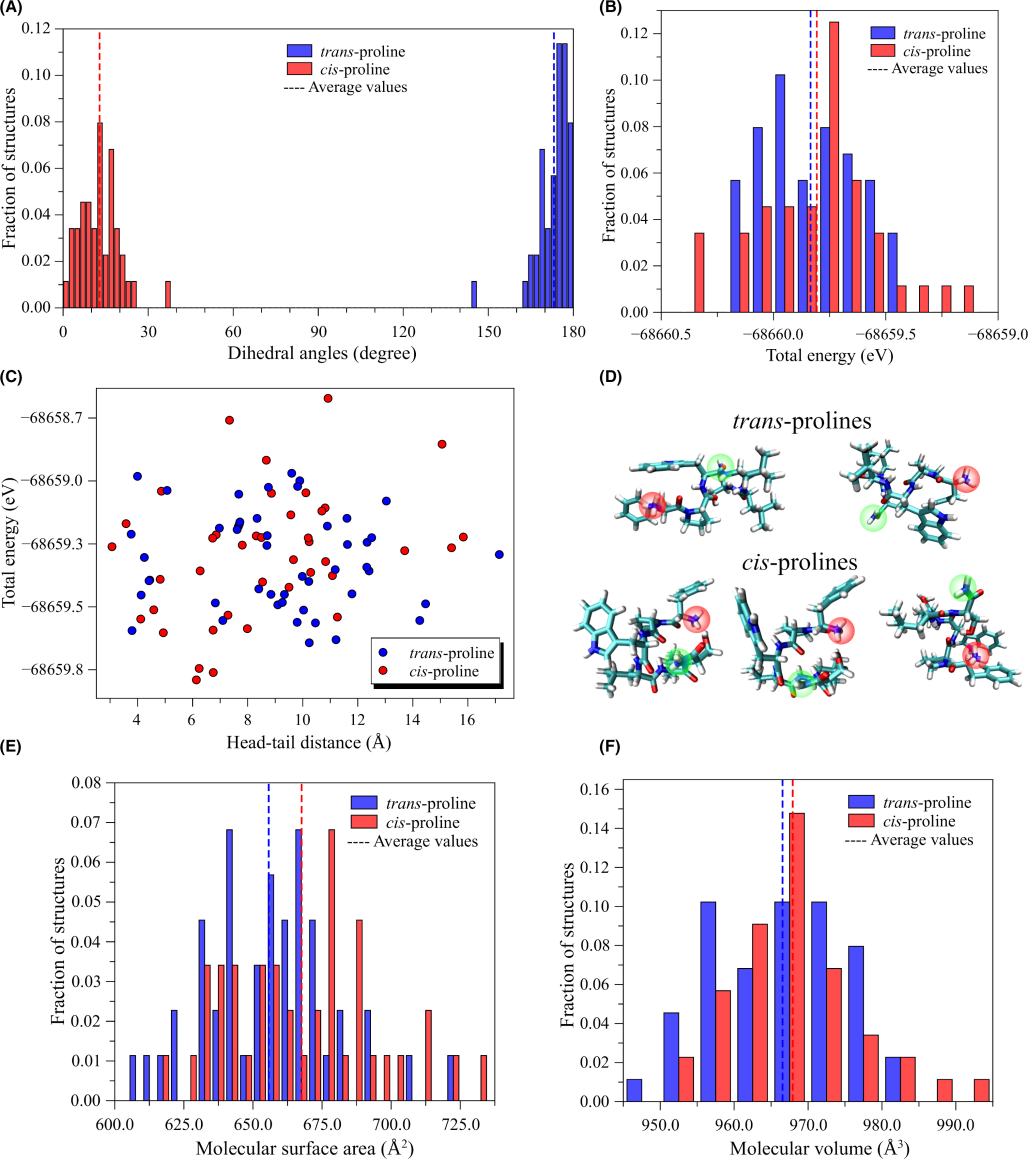
Comparison between PpT‐2 with *cis* and *trans* proline residues. (A) Distribution of the dihedral angles, (B) total energies, (C) total energy distributions as a function of head‐to‐tail distances, (D) structure of the most stable conformers, (E) molecular surface area and (F) molecular volume

To better interpret the dissimilarities noticed in the retention times of PpT‐2 fractions detected via chromatographic analysis (Figure 1H), a comparative study of structures containing *trans* and *cis* terminal proline was conducted. Figure 2A–F presents the relative distribution of structures (after geometry optimization) in relation to the dihedral angles of the P unit, molecular total energies, surface area and molecular volume.

Structures with dihedral angles close to 0º were defined as *cis*‐prolines, while those close to 180º are defined as *trans*‐prolines (Figure 2A). Note that *cis*‐prolines present slightly distorted structures, with dihedral angles *ca*. 13º, while average values close to 173º are noticed for *trans*‐prolines. Both systems present very similar energetic and structural features, which suggests that both the rotamers are expected to be present in the samples (Figure 2B–F). In particular, slightly lower energies are noticed for *cis*‐proline, which also present lower head‐to‐tail distances (Figure 2C,D). Peptides with terminal *cis*‐prolines also present slightly higher surface areas. Such subtle differences allow us to interpret the different retention times detected in the chromatographic analysis (Figure 1H). The involvement of peptides assuming *cis*‐*trans* isomerization of P was also investigated for bradykinin, a bioactive peptide found in vertebrates, including in skin secretion of amphibians.^51^ Bradykinin has three P residues, all crucial to establish the several conformations of the peptide,^52^ but only when all three residues assume the *trans* conformation, bradykinin is able to bind to its receptor.^53^ In this case, it promotes a range of pharmacological effects, mainly associated with inflammation, pain response, vasodilatation and blood pressure regulation.^54^ Given the energetical and structural similarities between both rotamers, the biological implication of the multiple conformations of PpT‐2 should be further investigated in the future.

### 3.3 Antimicrobial assays of PpT‐2

To explore biological activities of PpT‐2, antimicrobial assays against Gram‐negative and Gram‐positive bacteria were performed. Although most tryptophyllins described in the literature do not present antimicrobial activity,^55^ AcT‐2 (GMRPPWF‐NH_2_), found in the skin secretion of the red‐eyed leaf frog *Agalychnis callidryas*, inhibits the growth of *Staphylococcus aureus* (MIC = 256 μg/ml), *Escherichia coli* (MIC = 512 μg/ml) and *Candida albicans* (MIC = 128 μg/ml), that is, it has moderate antimicrobial action.^12^ However, for PpT‐2, no antimicrobial activity could be detected up to peptide concentrations of 1 mg/ml (Table S1).

### 3.4 Antioxidant properties of PpT‐2

Antioxidant compounds present an interesting capability of stopping or retarding oxidation chain reactions responsible for cell damage.^56^ The mechanism of action of these compounds generally involves a series of complex processes of free radicals deactivation/trapping via charge transfer (CT) mechanisms and/or chemical reactions.^51^ From a theoretical point of view, it is important to rank this activity for distinct compounds to identify optimized systems, which can be achieved via *in silico* studies.

**FIGURE 3:**
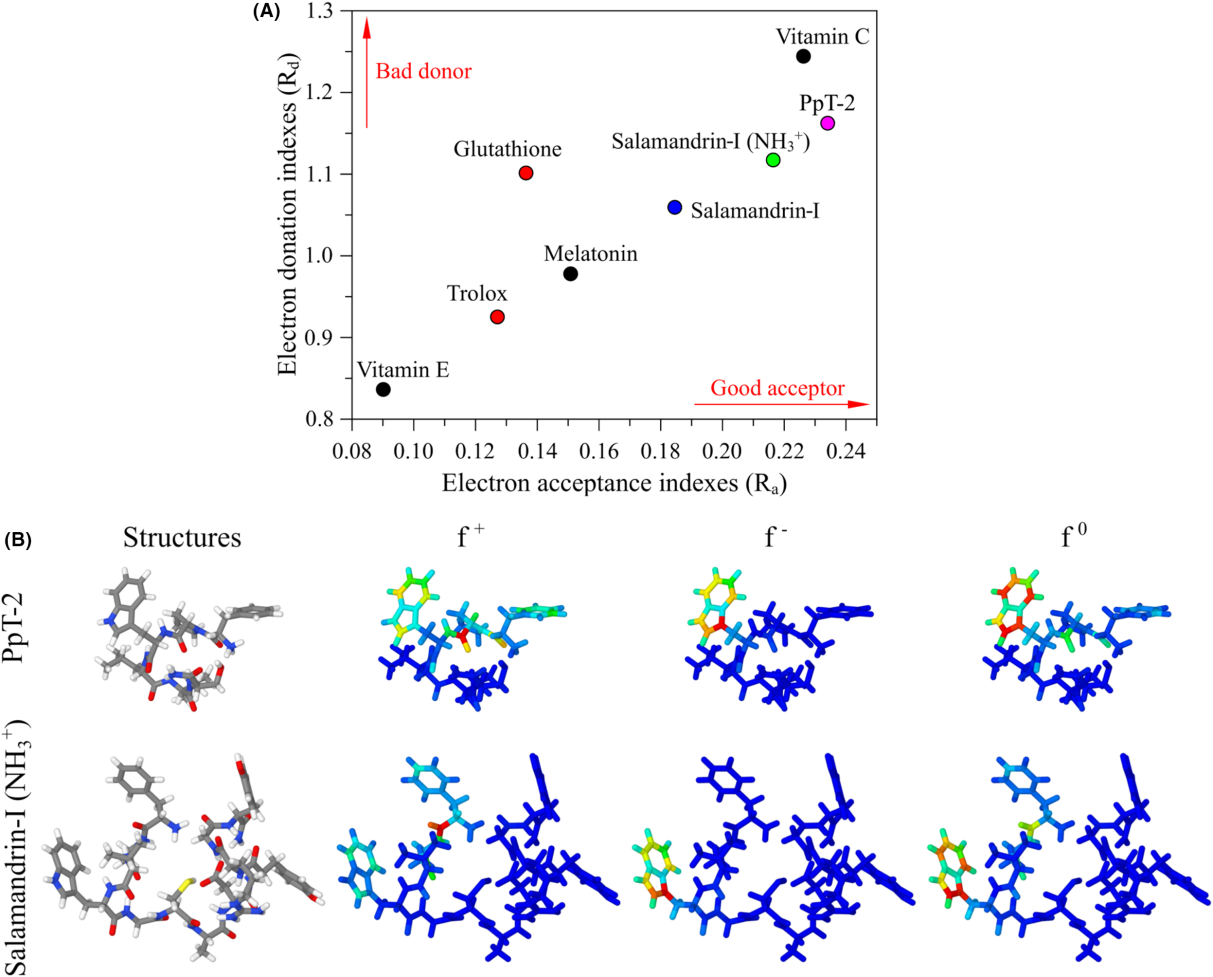
(A) Electron donation/acceptance ratio of PpT‐2 and protonated salamandrin‐I compared to literature values. (B) Molecular 3D representation of PpT‐2 and protonated salamandrin‐I, atom colour scheme: grey (C), red (O), blue (N) and white (H). Colour representation of Condensed‐to‐Atoms Fukui Indexes for reactions with nucleophiles (*f* ^+^), electrophiles (*f* ^−^) and free radicals (*f* ^0^). Red and blue colours represent reactive and non‐reactive sites, respectively. Other colours represent intermediate situations, following a RGB scale

In this context, quantum chemical calculations have been successfully employed to investigate relevant CT processes and antioxidant activities of biologic systems.^26^, ^40^, ^51^ The potential antioxidant properties of PpT‐2 (only the most stable conformer) were evaluated via DFT‐based calculations and compared with other antioxidants.^40^ The DAM map highlights PpT‐2 as a good electron acceptor and a bad electron donor, similarly to protonated salamandrin‐I (Figure 3A). This result suggests that these peptides might have similar antioxidant mechanisms. Note that the protonation at *N*‐terminus increases the acceptor properties of salamandrin‐I.^40^

**FIGURE 4:**
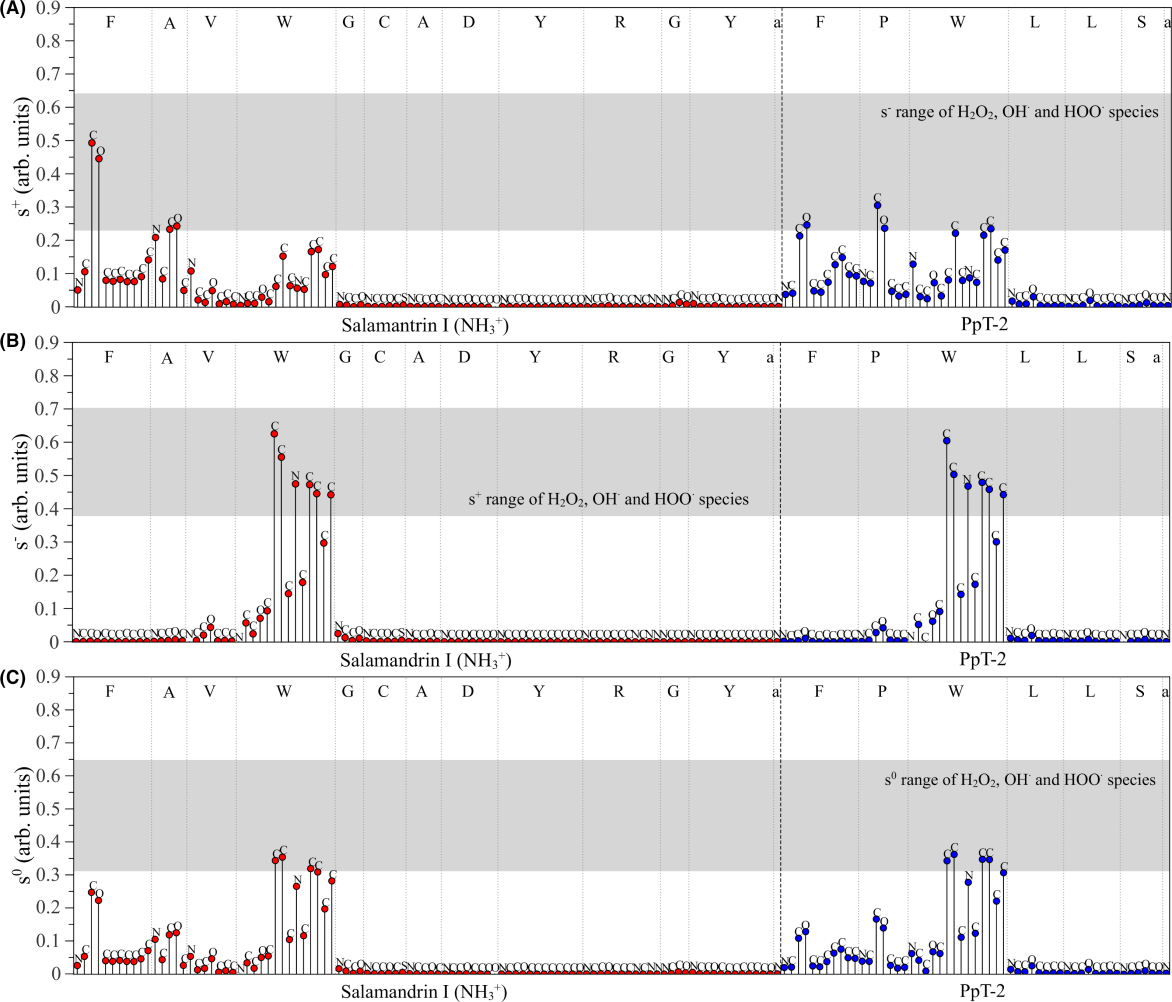
Comparative study of the local chemical softness of protonated salamandrin‐I and PpT‐2 in relation to H_2_O_2_, OH• and HOO•. Antioxidant activities: (A) antioxidants as electrophiles (*s* ^+^). (B) nucleophiles (*s* ^−^) and (C) free radicals’ scavengers (*s* ^0^)

Figure 3B presents a comparison between the CAFIs of salamandrin‐I and PpT‐2. It can be seen that the W unit plays an essential role on the reactivity of both the compounds, especially in relation to electrophiles (*f* ^−^) and free radicals (*f ^0^*). The reactivity towards nucleophiles (*f* ^+^) is spread over the structure, being more intense on the oxygen atoms of the terminal carboxamides. A comparison between the local softness of protonated salamandrin‐I (*N*‐terminal NH_3_^+^) and PpT‐2 is shown in Figure 4.

The shaded regions indicate the range associated with the local softness of typical oxidants (H_2_O_2_, OH^•^ and HOO^•^, estimated at the same level of theory by Plácido et al.,^40^) so that the similarities between *s* ^+^/*s* ^−^, *s* ^−^/*s* ^+^, and *s^0^*/*s^0^* indices of the peptides/common oxidative species can be compared. According to the hard–soft and acid–bases (HSAB) principle, chemical reactions are favoured between atoms with similar local softness; in this sense, atoms with *s* ^+^, *s* ^−^ and *s^0^* in the grey regions are supposed to interact effectively with the oxidant compounds.

In relation to the results reported by Plácido et al.,^40^ it can be noticed that the protonation of salamandrin‐I drives the *f* ^+^ reactivity (i.e. towards nucleophiles) to the terminal amino acid residues (phenylalanine (F) and alanine (A)), which is associated with the presence of a labile hydrogen (H) on the NH_3_^+^ (*N*‐terminal) group. For *f* ^+^, it is noticed the relevance of F, A and W amino acids for salamandrin‐I; and F, P and W for PpT‐2. For *f ^–^*, reactivity is dominated by W units in both peptides. As observed for *f ^0^* and *s^0^*, the reactivity of the systems towards free radicals is dominated by W, indicating the relevance of tryptophan on the antioxidant properties of the peptides. The two peptides present very similar local reactivities, reinforcing the hypothesis that they have similar mechanisms of action.

Different assays have been introduced to measure antioxidant capacity of biological molecules. The concept of antioxidant capacity first originated from chemistry and was later adapted to biology, medicine, epidemiology and nutrition.^57^ This concept provides a broader picture of the antioxidants present in a biological sample, as it considers the additive and synergistic effects of all antioxidants rather than the effect of single compounds. Therefore, it may be useful to study the potential health benefits of antioxidants on oxidative stress‐mediated diseases.^58^

The ability of PpT‐2 to scavenge ABTS and DPPH radicals *in vitro* was tested using trolox as the reference compound and the endogenous peptide glutathione as a reference antioxidant peptide (Table S2). PpT‐2 had an ABTS scavenging activity of 0.269 mg trolox equivalents per mg of peptide, which is comparable to that of other amphibian‐derived peptides, such as salamandrin‐I (0.285 mg trolox‐eq/mg peptide) and antioxidin‐RP1 (0.300 mg trolox‐eq/mg peptide). These values are higher than the activity of antioxidin‐I (0.010 mg trolox‐eq/mg peptide), but much lower than that of glutathione (1.911 mg trolox‐eq/mg peptide). Overall, the results indicate that PpT‐2 has free radical scavenging activities, the strongest activity being against ABTS radicals, and this ability is chemically associated with antioxidant activity. This agrees with previous studies, in which bioactive peptides identified in the skin secretion of amphibians had a strong antioxidant activity on ABTS and little or no activity on DPPH radicals, as reported for glutathione, salamandrin‐I, antioxidin‐RP1 and antioxidin‐I.^13^, ^40^

### 3.5 Cytotoxicity studies and *in vivo* toxicity of PpT‐2

**FIGURE 5:**
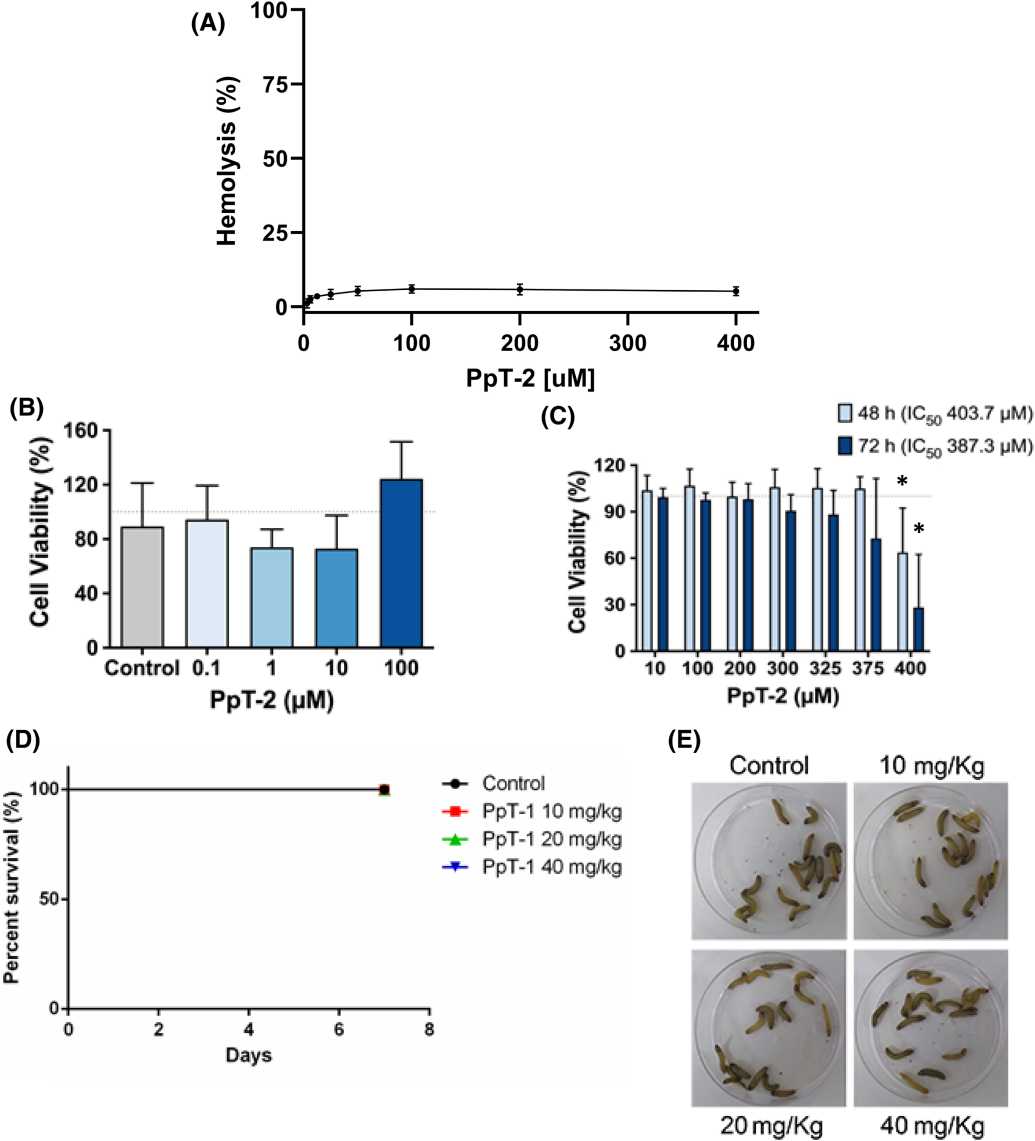
(A) Haemolytic activity determined for increasing concentrations of PpT‐2 against human RBCs. 0% and 100% haemolysis were defined based on the absorbance values obtained for the negative (buffer) and positive (Triton X‐100 0.1%) controls, respectively. Experiments were performed in triplicate. (B) Cytotoxicity studies in human microglial cells of PpT‐2 with concentrations ranging from 0.1 to 100 µM after 24 h of incubation. (C) Effect of PpT‐2 peptide on prostate cancer PC3 cell line viability with concentrations ranging from 10 to 400 µM after 48 h and 72 h of incubation. Data were normalized using the cell viability of control untreated cells, which was set to 100% and represented by the dashed grey line. The asterisk (*) denotes a significant difference compared with control untreated cells (*p* < 0.05, ordinary one‐way ANOVA followed by Dunnett’s multiple comparison test using untreated cells as the reference control groups for each time of incubation). IC_50_, half maximal inhibitory concentration. (D) *G*. *mellonella* log‐rank Mantel–Cox survival curve in the presence of different doses of PpT‐2 evaluated for seven days. Survival curves for *G*. *mellonella* larvae treated with 10, 20 and 40 mg/kg of PpT‐2. All larvae were injected with 10 μl of different doses of PpT‐2. Data from two experiments, *n* = 16 for all groups. (E) Representative larvae of groups, 0 and 7 days after treatment; the absence myelinization demonstrate the physiological good conditions

Despite the limitations of *in vitro* cytotoxicity assays, their importance in early drug development is unquestionable. There are several pros and cons of the use of cell viability or cytotoxicity assays as a reliable model of human medication.^59^ In this study, there was no statistically significant decrease in cell viability of human microglia treated with PpT‐2 (one‐way ANOVA; *F* (4, 10) = 2.074; *p* = 0.1593), as indicated by the resazurin assay up to 100 μM, suggesting an absence of cytotoxicity for this tryptophyllin (Figure 5B).

Microglia serve as the resident mononuclear phagocytes of the brain and are highly heterogeneous within a healthy central nervous system (CNS). The presence of activated glial cells localized in regions of brain injury is initially considered as a sign of pathology and, as such, considered for use as a sensitive marker to identify injury sites predestined for imminent tissue destruction.^60^ The absence of cytotoxicity *in vitro* (Figure 5B) suggests the possibility of neuroprotective studies to assess whether the intrinsic antioxidant activity of the molecule can lead to cellular neuroprotection through regulation of microglia activity.^13^ However, despite the intracellular effects caused by the peptide, such as modulation of the glutathione redox balance, studies on the internalization of this peptide still need to be performed.

The haemolysis assay is a rapid simple initial cytotoxicity assay. In general, peptides having high haemolytic activity are not suitable for therapeutic use. PpT‐2 demonstrated no significant haemolytic activity against human RBCs up to 400 µM (Figure 5A). The haemolytic assay has been used as a safety evaluation on the effect of peptides on a standardized model of mammalian cells. Wang et al.^17^ showed that an expanded tryptophyllin (AcT‐3, pEGKPYWPPPFLPE) from the skin of the red‐eye leaf frog *Agalychnis callidryas* also did not present haemolytic activity up to 160 μM for horse RBCs.

The anticancer potential of PpT‐2 was also evaluated through assessment of its antiproliferative effects on PC3 prostate cancer cells. The assay was performed in a serum‐free environment to avoid possible interactions. Despite no effect could be observed after 24 h of incubation (data not shown), an effect on PC3 cell viability after 48 and 72 h of incubation was recorded (Figure 5C), highlighting the antitumoral potential of PpT‐2. This tryptophyllin had antiproliferative effects after 48 and 72 h of incubation in comparison to untreated control cells, causing a decrease in cell viability at concentrations above 375 μM after 72 h of incubation, and this decrease was significant at 400 μM PpT‐2 for both time‐points.

The determined IC_50_ values were 403.7 μM and 387.3 μM for 48 and 72 h, respectively. These results are in agreement with similar effects observed, though at lower concentrations, for PsT‐1 against the same prostate cell line (PC3), a tryptophyllin from the skin secretion of the waxy monkey leaf frog *Phyllomedusa sauvagei*.^10^ There may be a link between the PpT‐2 antioxidant and antiproliferative effects. Tumour cells have higher levels of ROS than normal cells, due to their rapid proliferation rate.

Therefore, chemotherapeutic drugs could be specifically designed to promote or suppress the levels of ROS and consequently antagonize tumour progression. In an interesting study, a short peptide sequence (KRSH) was shown to display *in vitro* mitochondrial‐targeted antioxidant activity, inducing apoptosis in HeLa and MCF‐7 cancer cell lines, while drastically decreasing the levels of ROS. Despite the *in vitro* IC_50_ values of both tryptophyllins and KRSH not being very low, the relationship between ROS and metastasis, and the low toxicity of PpT‐2 makes it an appealing starting point for the future exploration of tryptophyllins for medium to long‐term treatment of certain tumour types.^61^

Despite the interest of an initial assessment of cytotoxicity *in vitro*, *in vivo* evaluation is often preferable. In this regard, *Galleria mellonella* larvae stand as an interesting *in vivo* model to evaluate microbial virulence, as well as the efficacy and toxicity of antimicrobials, showing results similar to those obtained with mammalian models.^62^ In the present work, none of the tested concentrations of PpT‐2 induced *G*. *mellonella* larvae death during the 7 days of treatment (Figure 5D,E). Thus, PpT‐2 presents low toxicity, similar to other peptides.^63^

### 3.6 Neuroprotective activity of the PpT‐2

Finally, the antioxidant capacity of PpT‐2 was also assessed in mammalian cells, namely mouse (*Mus musculus*) microglia that respond to tissue damage and infection by producing and releasing ROS and RNS to the surrounding CNS milieu.^64^ ROS are diffusible molecules capable of carrying out signal transduction processes in response to extracellular stimuli. A role of ROS in gliosis has been proposed from studies showing an inhibition by antioxidant treatment. The generation of ROS activates the inducible nitric oxide synthase (iNOS), enhancing nitric oxide (NO) production from glial and endothelial cells.^65^ Microglia‐induced ROS production and inflammation play an imperative role in neurodegenerative disorders such as Alzheimer's disease (AD) and Parkinson's disease (PD).^66^

**FIGURE 6:**
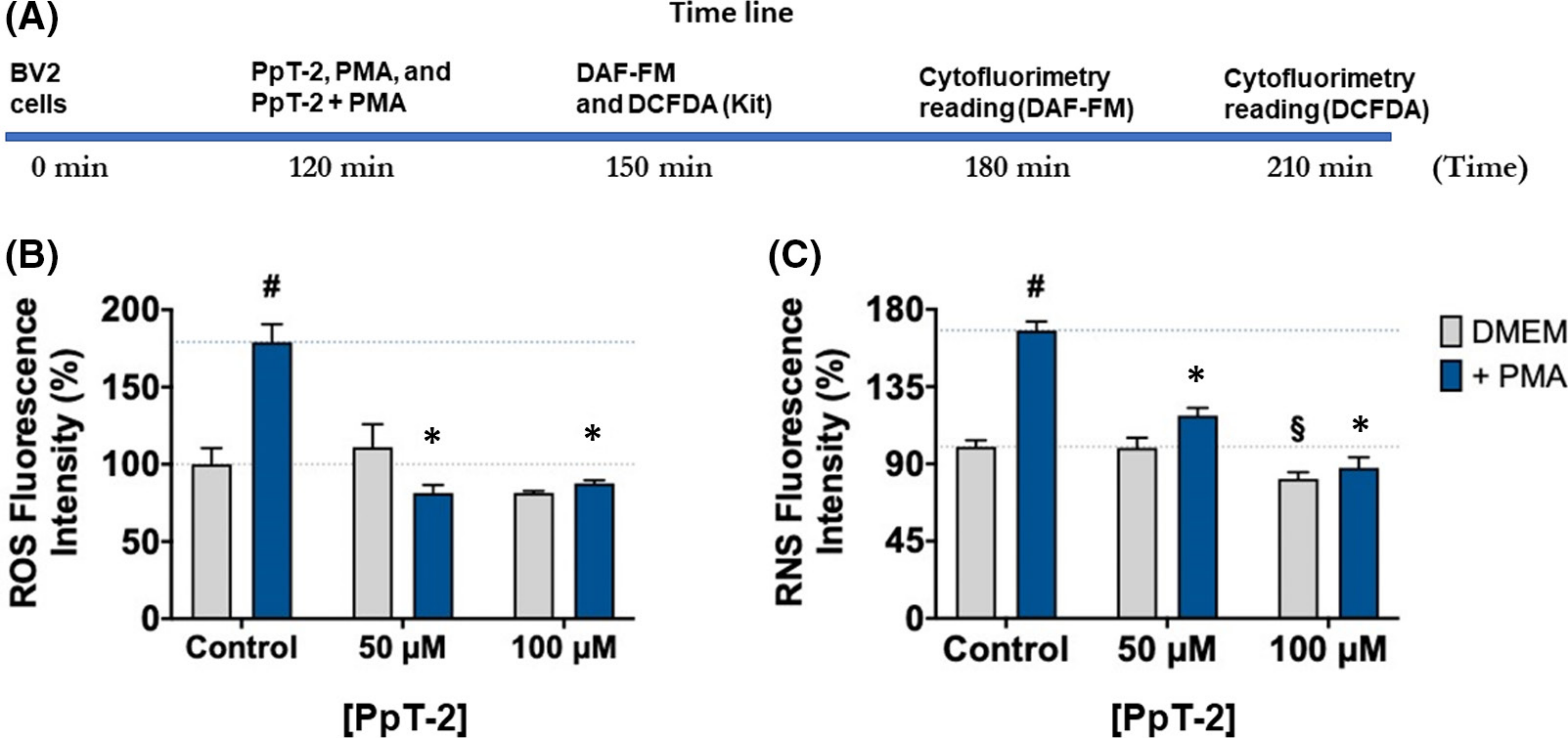
PpT‐2 displays antioxidant and neuroprotective properties in mouse microglial (BV‐2) cells. (A) Timeline of the assay. (B and C) Mouse BV‐2 cells microglia reactive oxygen species (ROS) and reactive nitrogen species (RNS) production were induced by phorbol 12‐myristate 13‐acetate (PMA) and treated by peptides at 50 and 100 µM. Data are shown as mean ± standard deviation. First, cells treated with PMA were compared with those maintained with medium only using an unpaired *t*‐test (the hash ‘#’ indicates a significant difference, *p* < 0.05). Then, an ordinary one‐way ANOVA was conducted, followed by Dunnett's multiple comparison tests, using untreated control cells (the section sign ‘§’ indicates a significant difference, *p* < 0.05) or PMA‐treated control cells (the asterisk ‘*’ indicates a significant difference, *p* < 0.05) as reference control group

Mouse microglial cells (BV‐2) were stimulated with PMA, a chemical known to induce ROS and RNS production,^67^ either in the presence or in the absence of PpT‐2 at two concentrations (Figure 6B,C). PMA activates protein kinase C, which, in turn, activates NADPH oxidase (NOX) by phosphorylation of p47phox, ultimately leading to increased ROS generation. Our results show that, at the concentrations tested, PpT‐2 exhibits potent inhibition of oxidative stress in BV‐2 during the 30 min of simultaneous incubation (peptide and PMA), that is, right at the beginning of treatment. Noteworthy, PpT‐2 inhibits the generation of RNS even in cells not stimulated by PMA, which means that inhibition occurs in these conditions in the basal state. Altogether, our results show that PpT‐2, through its antioxidant effects, controls the steady‐state levels of both ROS and RNS. Hence, this tryptophyllin holds promise as a therapeutic agent to treat or prevent neurodegenerative disorders given its antioxidant activity in microglia and the involvement of redox imbalance in the initiation and progression of Alzheimer's disease, Parkinson's disease, Huntington disease and others. Remarkably, despite the large number of tryptophyllins known to date, ours is the first study verifying this type of bioactivity and therapeutic potential for PpT‐2.

## 4 CONCLUSION

The Iberian green frog *P*. *perezi* secretes and stores on skin tissue a tryptophyllin‐like peptide, named PpT‐2, whose structure shows similarities to tryptophyllins of class 2, being an amidated peptide with a Pro‐Trp motif. *In silico* tests, performed to verify PpT‐2 antioxidant properties, revealed an electron donation/acceptance ratio similar to that of other antioxidants, being particularly similar to protonated salamandrin‐I. Both salamandrin‐I and PpT‐2 present a tryptophan residue important for their antioxidant properties, as indicated by *in silico* studies. *In vitro* experiments revealed that PpT‐2 acts as a free radical scavenger, mainly for the ABTS radical, and inhibits PMA‐induced oxidative stress in mouse microglia. Thus, *in vitro* results corroborate the *in silico* prediction of antioxidant activity. Additionally, PpT‐2 showed low cytotoxicity *in vivo* and *in vitro*, while displaying moderate antiproliferative effects against prostate cancer cells. Altogether, these results indicate that PpT‐2 stands as a promising peptide with potential therapeutic and biotechnological applications, mainly for the treatment or prevention of neurodegenerative disorders. This work also further demonstrates that amphibian skin secretions remain a valuable source of biological compounds of pharmacological and economic interest.

## CONFLICT OF INTEREST

The authors declare no conflicts of interest.

## AUTHOR CONTRIBUTIONS

**Alexandra Plácido:** Conceptualization (equal); formal analysis (equal); investigation (equal); writing – original draft (equal). **Constança Amaral:** Conceptualization (equal); formal analysis (equal); investigation (equal); writing – original draft (equal). **Catia Teixeira:** Conceptualization (equal); formal analysis (equal); investigation (equal). **Ariane Nogueira:** Formal analysis (equal); investigation (equal); writing – original draft (equal). **José Brango Vanegas:** Conceptualization (equal); formal analysis (equal); investigation (equal). **Eder Barbosa:** Conceptualization (equal); formal analysis (equal); investigation (equal); writing – original draft (equal). **Daniel C Moreira:** Data curation (equal); formal analysis (equal); investigation (equal); methodology (equal); visualization (equal); writing – original draft (equal); writing – review and editing (equal). **Amandda Carvalho:** Conceptualization (equal); formal analysis (equal); investigation (equal); writing – original draft (equal). **Maria Glória:** Conceptualization (equal); formal analysis (equal); investigation (equal); writing – original draft (equal). **Jhones Nascimento:** Formal analysis (equal); investigation (equal). **Patricia Albuquerque:** Conceptualization (equal); formal analysis (equal); investigation (equal); resources (equal); supervision (equal). **Felipe Saldanha‐Araújo:** Conceptualization (equal); formal analysis (equal); investigation (equal); resources (equal); supervision (equal); writing – original draft (equal). **FIlipe Lima:** Conceptualization (equal); formal analysis (equal); investigation (equal); visualization (equal); writing – original draft (equal). **Augusto Batagin‐Neto:** Conceptualization (equal); formal analysis (equal); investigation (equal); visualization (equal); writing – original draft (equal). **Selma Kuckelhaus:** Conceptualization (equal); formal analysis (equal); investigation (equal); resources (equal); supervision (equal); writing – original draft (equal). **Lucinda Bessa:** Conceptualization (equal); formal analysis (equal); investigation (equal); writing – original draft (equal). **Jaime Freitas:** Conceptualization (equal); formal analysis (equal); investigation (equal); writing – original draft (equal). **Guilherme Brand:** Conceptualization (equal); formal analysis (equal); investigation (equal); writing – original draft (equal). **Nuno Santos:** Conceptualization (equal); formal analysis (equal); investigation (equal); writing – original draft (equal). **João Relvas:** Conceptualization (equal); formal analysis (equal); investigation (equal); writing – original draft (equal). **Paula Gomes:** Conceptualization (equal); formal analysis (equal); investigation (equal); writing – original draft (equal); writing – review and editing (equal). **José Leite:** Conceptualization (equal); formal analysis (equal); investigation (equal); supervision (equal); writing – original draft (equal); writing – review and editing (equal). **Peter Eaton:** Conceptualization (equal); formal analysis (equal); investigation (equal); writing – original draft (equal).

## Supporting information

Supplementary Material

## Data Availability

The data that support the findings of this study are available from the corresponding author upon reasonable request.
