# Supplementary material for: Neuroprotective effects on microglia and insights into the structure–activity relationship of an antioxidant peptide isolated from Pelophylax perezi
Source: J Cell Mol Med. 2022 Apr 23;26(10):2793–807. doi: 10.1111/jcmm.17292 (PMC9097852; doi:10.1111/jcmm.17292)
Supplement: Supplementary file 1 — Supplementary Material [file JCMM-26-2793-s001.docx]

**Support Information (SI)**

**Neuroprotective effects on microglia and insights into the structure-activity relationship of an antioxidant peptide isolated from *Pelophylax perezi***

Alexandra Plácido^1,a,*^, Constança Pais do Amaral^2,a^, Cátia Teixeira^1^, Ariane Nogueira^3^, José Brango-Vanegas^3^, Eder Alves Barbosa^3,4^, Daniel C. Moreira^3^, Amandda É. Silva-Carvalho^5^, Maria da Gloria da Silva^3^, Jhones do Nascimento Dias^6,7^, Patrícia Albuquerque^6,8^, Felipe Saldanha-Araújo^5^, Filipe Camargo D. A. Lima^9^, Augusto Batagin-Neto^10^, Selma Kuckelhaus^3^, Lucinda J. Bessa^1,11^, Jaime Freitas^12^, Guilherme Dotto Brand^4^, Nuno C. Santos^2^, João B. Relvas^13^, Paula Gomes^1^, José Roberto S. A. Leite^1,3^, Peter Eaton^1,14^

^1^LAQV/REQUIMTE, Department of Chemistry and Biochemistry, Faculty of Sciences of the University of Porto, Porto, Portugal;

^2^Instituto de Medicina Molecular, Faculdade de Medicina, Universidade de Lisboa, Lisbon, Portugal;

^3^Center for Research in Applied Morphology and Immunology (NuPMIA), University of Brasilia, Brasilia, Brazil;

^4^Laboratory of synthesis and analysis of biomolecules (LSAB), Institute of Chemistry (IQ), University of Brasilia, Brasília, Brazil;

^5^Laboratory of Hematology and Stem Cells, Faculty of Health Sciences, University of Brasilia, Brasília, Brazil;

^6^Department of Cell Biology, Institute of Biological Sciences, University of Brasília, Brasília, Brazil;

^7^Biomedicine Course, Federal University of Delta do Parnaíba (UFDPar), Parnaíba, Brazil;

^8^Faculty of Ceilândia, University of Brasilia, Brasilia, Brazil;

^9^Federal Institute of Education, Science and Technology of São Paulo, Matão, Brazil;

^10^São Paulo State University (UNESP), Campus of Itapeva, Itapeva/SP, Brazil;

^11^Egas Moniz interdisciplinary research Center (CiiEM), Egas Moniz - Cooperative for Higher Education, CRL, Almada, Portugal;

^12^Institute for Research and Innovation in Health (i3S), National Institute of Biomedical Engineering (INEB), University of Porto, Porto, Portugal;

^13^Institute for Research and Innovation in Health (i3S), Institute for Molecular and Cell Biology (IBMC), University of Porto, Porto, Portugal;

^14^The Bridge, School of Chemistry, Joseph Banks Laboratories, University of Lincoln, Lincoln, UK.

^a^ These authors equally contributed to this work.

^*^**Correspondence**: [alexandra.nascimento@fc.up.pt](mailto:alexandra.nascimento@fc.up.pt), alexandra.placido@gmail.com; Phone number: +351 220402000 (A.P.). Department of Chemistry and Biochemistry, Faculty of Sciences of the University of Porto. Rua do Campo Alegre, s/n, 4169-007 Porto, Portugal.

**Material and Methods**

**Biological samples.** *Pelophylax perezi* specimens were manually captured in São Miguel Island, located in the Azores archipelago, Portugal, under the license n° 15/2019/DRA (Regional Directorate for the Environment, Government of Azores, Portugal) (**Figures 1A** and **1B**). The cutaneous secretion from *P. perezi* was obtained by electrical stimulation (6 V), collected in Milli-Q water in 50 mL tubes, filtered (Millipore filters, 0.22 μm), immediately frozen and lyophilized.^1^

**Skin morphology.** Skin tissue samples were taken and preserved in 10% neutral formaldehyde solution. To prepare the blocks, samples were subjected to washing and dehydration in ascending solutions of ethanol (70% to absolute). Tissue was cleared in xylene and was routinely embedded in paraffin. The blocks were cut into 6 μm sections and stained with hematoxylin and eosin (H&E), to evaluate tissue morphology, and periodic acid-Schiff’s (PAS), to stain polysaccharides. All staining protocols were performed according to standard laboratory protocols.^2^

**Mass spectrometry**. Dried fractions were redissolved in Milli-Q water. The amount of solvent ranged from 10 to 100 μL and was adjusted according to the UV absorbance obtained for each fraction. One-microliter aliquots of the chromatographic fractions were dissolved in α-cyano-4-hydroxycinnamic acid (ACH) matrix solution (1:3, v/v) and then applied on the plate for subsequent analysis by matrix-assisted laser desorption ionization/time of flight mass spectrometry (MALDI-TOF MS).

The molecular mass of the peptides was determined by MALDI TOF MS using an UltraFlex Xtreme mass spectrometer (Bruker Daltonics, Bremen, Germany) in the positive reflected mode, controlled by FlexControl software. The ions of interest were fragmented in the LIFT mode (MS/MS) for *De Novo* sequencing. Before each analysis, the spectrometer was calibrated using a mixture of peptides. A purified chromatographic fraction of the peptide was submitted to automatic sequencing by Edman degradation, resolving the L/I ambiguities and confirming the amino acid sequence. The new tryptophyllin-like sequence was compared with all peptides found in skin secretions of other frogs reported in the literature and relevant databases. ProParam software was used for computation of physical and chemical parameters.^3^

**In silico analysis** (**continuation**). Preliminary geometry optimizations were conducted for all the conformers in a Hartree–Fock (HF) approach using the semiempirical Hamiltonian PM6 implemented in the MOPAC2016 computational package, with solvent effects (water) modelled by conductor like-screening model (COSMO). All the geometries were subsequently fully optimized in the framework of Kohn–Sham density functional theory (KS-DFT) using Becke’s three-parameter hybrid exchange functional with the Lee–Yang–Parr correlation functional (B3LYP), and 6-31G(d) basis set on all the atoms. The presence of the solvent in KS-DFT calculations was simulated via polarizable continuum model (PCM). The average values of dihedral angles, total energies, surface area, volume, and head-to-tail distances were evaluated for the optimized systems via a specifically designed Fortran 90 routine. For comparison purposes, the structure of protonated (N-terminus) salamandrin-I peptide was also designed and optimized at the same level of theory.^4^ Condensed-to-atoms Fukui indices (CAFI),^5-10^ local softness (s_k_^+^, s_k_^–^ and s_k_^0^) and antioxidant properties were evaluated considering the most stable geometry of PpT-2, at the same theoretical level of the geometry optimizations, and following previously reported approaches.

The antioxidant properties of PpT-2 were compared to those of salamandrin-I, glutathione,^4^ Trolox, and other antioxidant compounds, considering the electron acceptance (R_a_) and electron donation (R_d_) indices in terms of donor-acceptor map (DAM).^11^ KS-DFT based calculations were conducted with the aid of the Gaussian 09 computational package.

**Peptide synthesis**

*Method 1*: PpT-2 (FPWLLS-NH_2_) was manually synthesized using the Fmoc/tert-butyl method. For further details on the methodology used for peptide purification and mass spectrometric analysis to confirm purity and primary structure, please refer to previous publications. Measuring peptide concentration: [Peptide concentration] mg/mL = (A_280_ × DF × M_w_)/ε (where A_280_ is the absorbance of the peptide solution at 280 nm in a 1 cm cell, DF is the dilution factor, M_w_ is the molecular weight of the peptide, and ε is the molar extinction coefficient of tryptophan or tyrosine at 280 nm).

*Method 2*: PpT-2 was similarly assembled by the standard Fmoc/tBu orthogonal protection scheme on a Symphony X peptide synthesis instrument (Gyros Protein Technologies, Tucson, AZ, USA), following in-house methods. Briefly, the peptide chain was grown in the *Ct→Nt* direction on a Fmoc-Rink-amide MBHA resin 100-200 mesh and 0.52 mmol/g functionalization (NovaBiochem) via alternating (i) deprotection (removal of the *N*^α^-Fmoc temporary protecting group with 20% piperidine in dimethylformamide, from Sigma-Aldrich, Lisbon, Portugal) and (ii) amino acid coupling (addition of the *N*^α^ -Fmoc-protected amino acid residue activated *in situ* with *O*-(1*H*-6-chlorobenzotriazole-1-yl)-1,1,3,3-tetramethyluronium hexafluorophosphate (HCTU) in the presence of *N*-ethyl-*N*,*N*-diisopropylamine (DIEA), VWR International, Amadora, Portugal) steps.

Once assembled, the peptide was cleaved from the resin with concomitant removal of all side chain protecting groups, by a 2 h acidolysis at room temperature, using a cleavage cocktail containing TFA, TIS (Sigma-Aldrich) and deionized water (95:2.5:2.5, v/v/v) in the proportion of 1 mL cocktail per 100 mg of peptidyl-resin. After the resin was removed by suction filtration, the crude peptide was precipitated from the filtrate using cold tert-butylmethyl ether (MTBE, Sigma-Aldrich), and isolated by centrifuged at 0ºC and 3,500 rpm for 10 minutes. The supernatant was discarded, and the peptide pellet was resuspended in fresh MTBE and the centrifugation step was repeated. This procedure was done for three times more, after which the peptide pellet was left in a vacuum desiccator overnight, and next solubilized with 0.1 M aqueous acetic acid.

The crude peptide was purified by preparative reverse-phase high performance liquid chromatography (RP-HPLC) on a Merck-Hitachi LaPrep Sigma system (VWR International, Radnor, Pennsylvania, USA), using a 0 to 100% linear gradient of ACN in 0.05% aqueous TFA. A final purity degree of about <99% was determined by analytical RP-HPLC on a Merck-Hitachi LaChrom Elite instrument (Radnor, Pennsylvania, USA). The structure of the target peptide was confirmed by electrospray ionization-ion trap mass spectrometry (ESI-IT MS) analysis on a Thermo Finnigan LCQ DECA XP system (Thermo Fisher Scientific), with observation of peaks associated to the quasi-molecular ions of the peptide and its dimer, at m/z values of 761.80 and 1522.27 a.m.u., respectively. The pure peptide was quantitated by microvolume spectrophotometry, on a NanoDrop One (Thermo Scientific). Finally, the peptide was freeze-dried in a VirTis BenchTop Pro 9L instrument and stored at -20ºC until further use.

**ABTS and DPPH details**. The ABTS assay was performed by following the procedure of Gião and co-workers.^12^ Briefly, to oxidize the colourless ABTS to the blue-green ABTS^+^ radical cation, ABTS (7 mM) was mixed with ammonium persulfate (2.45 mM) and kept for 12–16 h at room temperature (RT) in the dark. Then, the ABTS^+^ solution was diluted in water to an absorbance of 0.70 at 734 nm (Shimadzu 1240 UV-visible spectrophotometer). Aliquots (10 μL) of PpT-2 and 6-hydroxy-2,5,7,8-tetramethylchroman 2-carboxylic acid (Trolox) were mixed with 190 μL of ABTS^+^ solution and the absorbance at 734 nm was read after 6 min of the onset of the reaction. The decrease (%) in absorbance at 734 nm, which corresponds to the concentration of ABTS^+^, caused by PpT-2 was compared with that of a standard curve built with different concentrations (2–64 μg/mL) of Trolox. The experiment was performed in triplicates. Results were expressed as mg of Trolox equivalents/mg peptide.

The DPPH scavenging activity of PpT-2 was measured using the method described by Marxen and collaborators. A stock solution of DPPH^●^ was prepared in ethanol at 60 μM with an absorbance of 0.7 at 515 nm (Shimadzu 1240 UV–visible spectrophotometer). Aliquots of 20 μL of PpT-2 and Trolox were mixed with 180 μL of DPPH ethanolic solution and the absorbance at 515 nm was read after 30 min of the onset of the reaction. The decrease (%) in absorbance at 515 nm, which corresponds to the concentration of DPPH, caused by PpT-2 was compared with that of a standard curve built with different concentrations (2–64 μg/mL) of Trolox. The experiment was performed in triplicates. Results were expressed as mg of Trolox equivalents/mg peptide.

**Tables**

**Table S1**. Minimum inhibitory concentration (MIC) of synthetic peptide PpT-2 against four ATCC reference bacterial strains. Experiments performed in triplicate.

| **Peptide** | **MIC (µg/mL)** | | | |
| --- | --- | --- | --- | --- |
|  | *E. coli*  ATCC 25922 | *P. aeruginosa* ATCC 27853 | *S. aureus*  ATCC 25923 | *E. faecalis* ATCC 29212 |
| PpT-2 | ˃ 1024 | ˃ 1024 | ˃ 1024 | ˃ 1024 |

**Table S2.** Radical scavenging activity of PpT-2 and other amphibian-derived peptides according to different *in vitro* antioxidant assays. Results are expressed as mg of trolox equivalent per mg of peptide and presented as mean ± standard deviation from three independent experiments.

| **Peptide** | ***In vitro* antioxidant assays (Trolox-eq/mg)** | |
| --- | --- | --- |
|  | **ABTS** | **DPPH** |
| PpT-2 | 0.269 ± 0.002 | 0.001 ± 0.001 |
| Antioxidin-RP1¹ | 0.300 ± 0.002 | 0.053 ± 0.003 |
| Antioxidin-I¹ | 0.010 ± 0.009 | 0.002 ± 0.000 |
| Salamandrin-I² | 0.285 ± 0.003 | 0.081 ± 0.005 |
| Glutathione² | 1.911 ± 0.003 | 0.829 ± 0.005 |

ABTS, 2,2-azino-bis(3-ethylbenzothiazoline-6-sulphonic acid); DPPH, 2,2-diphenyl-1-picrylhydrazyl.

¹Data from Barbosa et al. (2018).

²Data from Plácido et al. (2020).

**References (SI)**

1. Barbosa EA, Oliveira A, Plácido A, et al. Structure and function of a novel antioxidant peptide from the skin of tropical frogs. *Free Radic Biol Med*. 2018; **115**: 68-79. doi:10.1016/j.freeradbiomed.2017.11.001
2. Luna LG. Manual of Histologic Staining Methods of the Armed Forces Institute of Pathology. New York: McGraw-Hill Book Company; 1970; **3**(3): 249. doi:10.1016/s0031-3025(16)39410-7.
3. Gasteiger E, Hoogland C, Gattiker A. et al. Protein identification and analysis tools on the ExPASy server. *The Proteomics Protocols Handbook*. 2005; 571-607. DOI: 10.1385/1-59259-890-0:571
4. Plácido A, Bueno J, Barbosa EA, et al. The Antioxidant Peptide Salamandrin-I : First Bioactive Peptide Identified from Skin Secretion of Salamandra Genus (*Salamandra salamandra*). *Biomolecules*. 2020; **10**(4): 512. doi:10.3390/biom10040512
5. Klamt A, Schüürmann G. COSMO: A new approach to dielectric screening in solvents with explicit expressions for the screening energy and its gradient. *J Chem Soc Perkin Trans 2*. 1993; (5): 799-805. doi:10.1039/P29930000799
6. Becke AD. Density-functional thermochemistry. III. The role of exact exchange. *J Chem Phys*. 1993; **98**(7): 5648-5652. doi:10.1063/1.464913
7. Cancès E, Mennucci B, Tomasi J. A new integral equation formalism for the polarizable continuum model: Theoretical background and applications to isotropic and anisotropic dielectrics. *J Chem Phys*. 1997; **107**(8): 3032-3041. doi:10.1063/1.474659
8. Bellver-Landete V, Bretheau F, Mailhot B, et al. Microglia are an essential component of the neuroprotective scar that forms after spinal cord injury. *Nat Commun*. 2019; **10**(1): 518. doi:10.1038/s41467-019-08446-0
9. Yang W, Mortier WJ. The Use of Global and Local Molecular Parameters for the Analysis of the Gas-Phase Basicity of Amines. *J Am Chem Soc*. 1986; **108**(19): 5708-5711. doi:10.1021/ja00279a008
10. Frisch MJ, Trucks GW, Schlegel HB, et al. Gaussian 09, Revision D.01. Gaussian, Inc., Wallingford CT, 2009.
11. Martínez A, Rodríguez-Gironés MA, Barbosa A, Costas M. Donator acceptor map for carotenoids, melatonin and vitamins. *Phys Chem A*. 2008; **112**(38): 9037–9042. doi: 10.1021/jp803218e
12. Gião MS, González-Sanjosé ML, Rivero-Pérez MD, et al. Infusions of Portuguese medicinal plants: Dependence of final antioxidant capacity and phenol content on extraction features. *J Sci Food Agric*. 2007; **87**(14): 2638-2647. doi:10.1002/jsfa.3023
